# How to Operate on a Huge Intrathoracic Mass? A Practical New Classification and a Special Innovative Surgical Method for the Operation of Huge Intrathoracic Masses

**DOI:** 10.1002/ccr3.72696

**Published:** 2026-05-10

**Authors:** Mohammad Reza Farahnak, Muhammad Sina Salimikouchi, Mohammad Javad Najafzadeh, Amir Baniasad

**Affiliations:** ^1^ Department of Internal Medicine, Air Pollution and Respiratory Diseases Research Center Ahvaz Jundishapur University of Medical Sciences Ahvaz Iran; ^2^ Department of Medicine Kerman University of Medical Sciences Kerman Iran; ^3^ Lung Disease Research Center Mashhad University of Medical Sciences Mashhad Iran

**Keywords:** chest wall surgery, mediastinal masses, rib resection, thoracic masses, thoracotomy, tumor removal

## Abstract

Huge intrathoracic masses occupying over half of the hemithorax and adhering to multiple thoracic surfaces can be safely resected using a surface‐based classification and targeted rib resection strategy, which enables optimal access, avoids fragmentation, and facilitates en‐bloc tumor removal.

## 1 Introduction

Surgical management of large intrathoracic masses presents significant challenges due to limited access to the thoracic cavity, close proximity to vital structures, and potential adhesions to surrounding tissues. Achieving safe exposure and complete resection is critical, particularly for lesions that occupy most of the hemithorax or involve multiple thoracic surfaces [1, 2].

Intrathoracic masses can arise from various thoracic structures and include both benign and malignant histology [3, 4]. While minimally invasive approaches such as video‐assisted thoracic surgery (VATS) or robotic‐assisted surgery are useful for selected lesions, most large masses require open thoracotomy for safe removal [5, 6].

To guide surgical planning, we applied a volume‐ and surface‐based classification system that categorizes intrathoracic masses according to their proportion of hemithorax volume and the number of surfaces involved (small, medium, large, very large, and huge). Lesions defined as huge intrathoracic masses (HITM) occupy more than 50% of the hemithorax and adhere to three or more major thoracic borders (diaphragm, mediastinum, lung apex, and chest wall). This framework helps distinguish cases that can be managed with standard thoracotomy from those requiring modified approaches for optimal exposure.

Tumor volume was estimated using CT imaging by calculating the lesion area on sequential slices based on pixel measurements. The total volume was then derived using the Cavalieri principle by summing cross‐sectional areas and multiplying by slice thickness. This approach enabled assessment of tumor size as well as its spatial relationship with adjacent thoracic structures.

For HITM, we employed a specialized thoracotomy technique that identifies the rib adjacent to a free edge of the mass (or, when no free edge exists, at the most voluminous portion) to enter the thoracic cavity safely, release adhesions, and achieve complete resection without fragmentation. In this report, we present three cases of HITM successfully managed using this systematic approach, demonstrating how careful preoperative planning and tailored surgical access can facilitate safe en‐bloc removal and favorable early outcomes.

## 2 Case 1

### 2.1 Case History

A 4‐year‐old boy with a chief complaint of non‐productive cough and a history of multiple respiratory tract infections over approximately 2 months was referred to our tertiary hospital. Vital signs on admission were: pulse rate (PR) 94/min, blood pressure 110/70 mmHg, respiratory rate (RR) 26/min, and temperature 36.8°C. Physical examination revealed decreased breath sounds over the right lung and mild scoliosis. Initial evaluations, including laboratory testing and chest radiography, were performed. Laboratory results showed a white blood cell (WBC) count of 5600/μL and a hemoglobin (Hb) concentration of 11.3 mg/dL.

**FIGURE 1:**
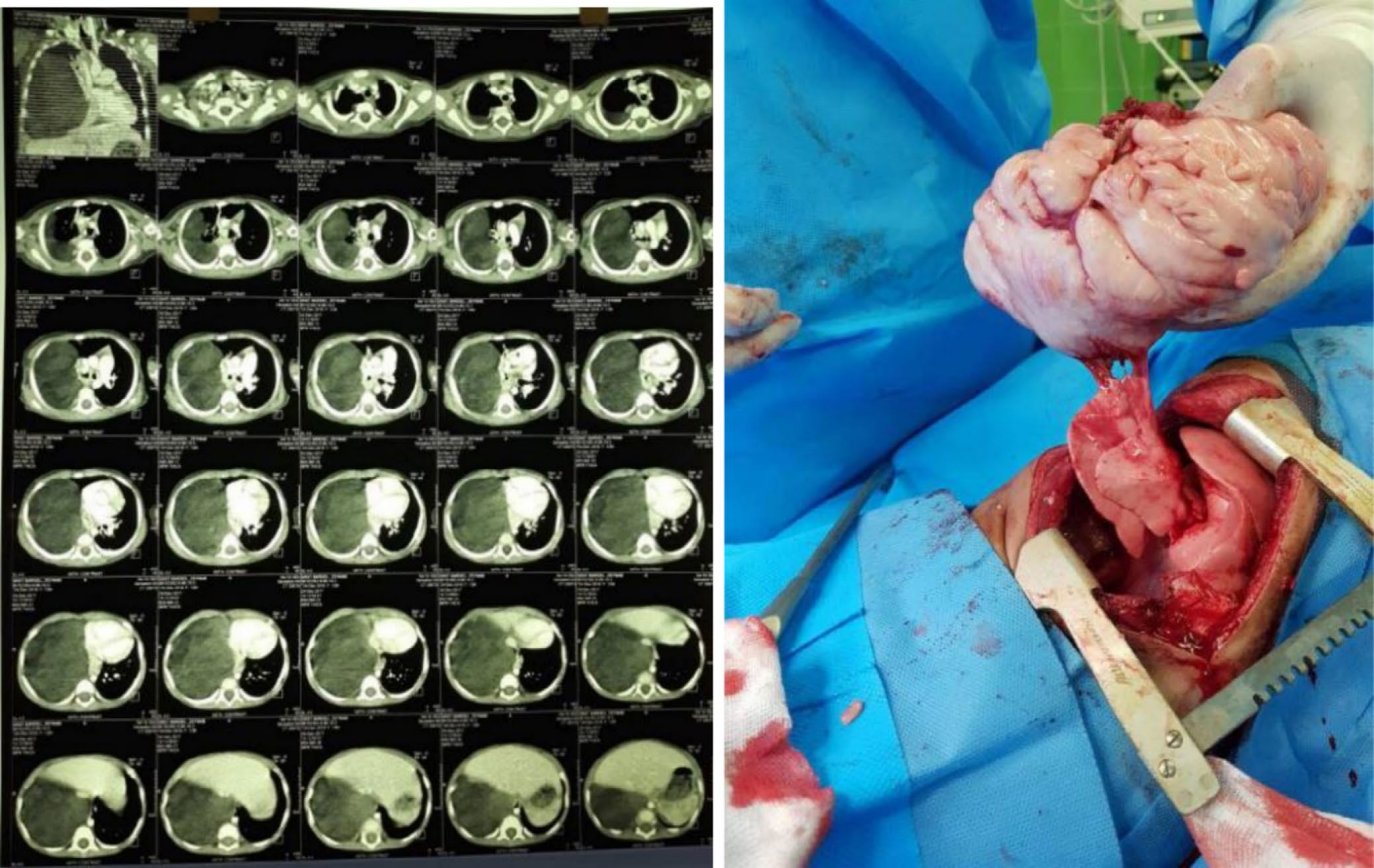
Left picture, lung CT scan of a 4‐year‐old boy with a huge intrathoracic mass. The right picture shows that the huge myolipoma mass was completely resected by our surgical method.

Chest X‐ray demonstrated a space‐occupying mass in the right hemithorax, causing mediastinal shift to the left and leftward convexity of the vertebral column. A chest CT scan was performed, and based on our proposed classification, the lesion was categorized as HITM (Figure 1). Sono‐guided core needle biopsy was compatible with myolipoma.

### 2.2 Differential Diagnosis

Lipoma, Teratoma, Lipoblastoma, Liposarcoma, Myolipoma.

### 2.3 Outcome and Follow‐Up

The patient underwent a right posterolateral thoracotomy. Since the superior border of the mass was free, the fourth intercostal space was initially identified; however, entry into the thoracic cavity was not possible via this route. Consequently, the fifth rib was resected to obtain access. Adhesions surrounding the mass were released. The medial aspect of the mass was attached to the mediastinum, and multiple ligations were performed using silk sutures in this area. The mass was then completely excised. A chest tube was placed, and the thorax was closed. The operative time was 2.5 h. No blood transfusion was required, and no postoperative complications occurred.

Postoperatively, the patient was transferred to the ICU. After 24 h, he was moved to the ward, where oral intake was initiated. He was discharged on postoperative day 5 in good general condition.

## 3 Case 2

### 3.1 Case History

A 6‐year‐old girl with frequent coughing, shortness of breath, and wheezing was referred to our tertiary hospital. Her symptoms had worsened over the previous month. On admission, her vital signs were: pulse rate (PR) 90/min, blood pressure 110/70 mmHg, respiratory rate (RR) 30/min, and temperature 36.9°C. On physical examination, decreased breath sounds and wheezing were heard in the mid and upper thirds of the right hemithorax. Laboratory results showed a white blood cell (WBC) count of 11,000/μL and a hemoglobin level of 10.5 mg/dL.

**FIGURE 2:**
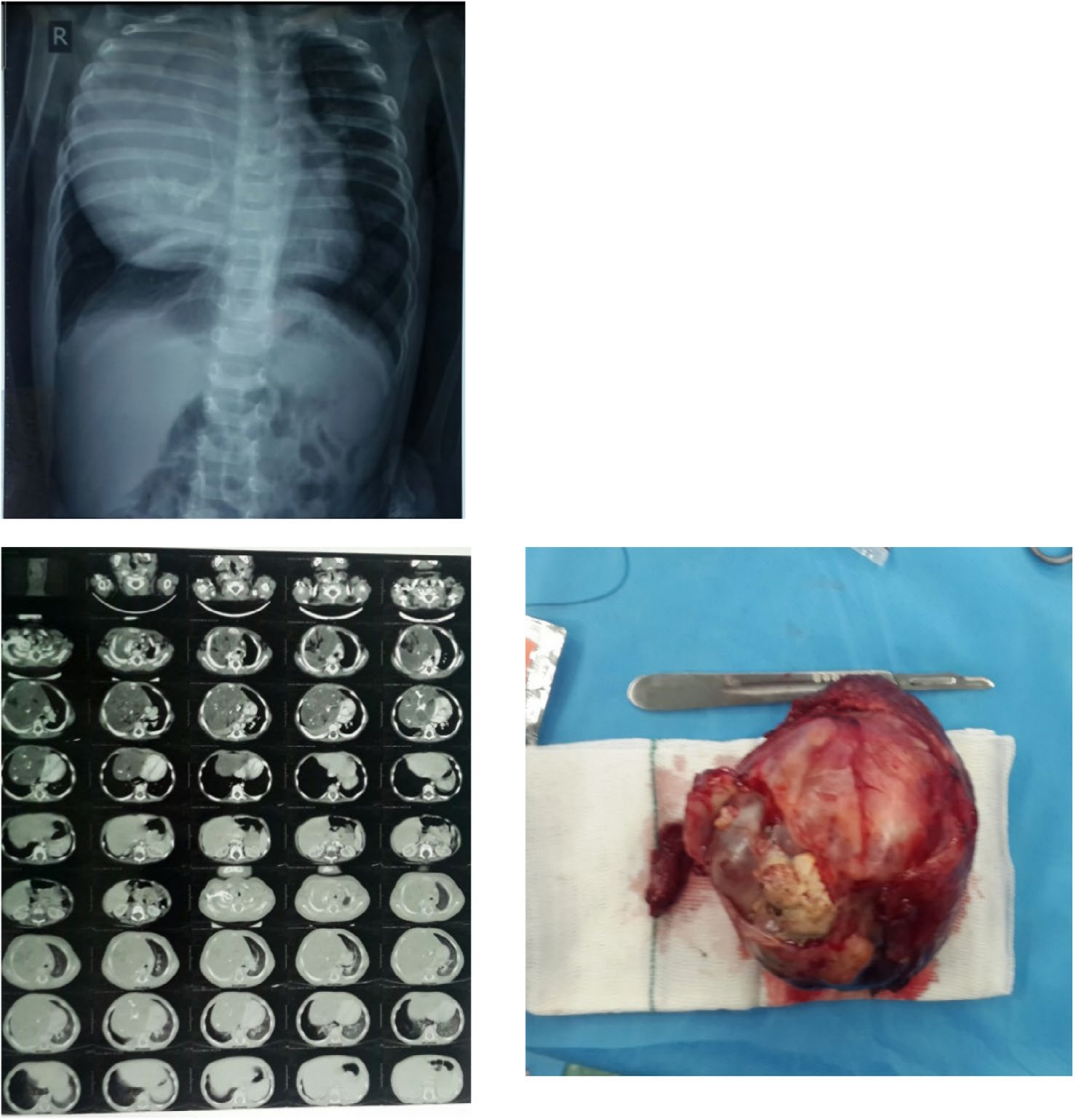
Chest X‐ray (left), lung CT scan (middle) of a huge intrathoracic mass, which was extracted by our method of surgery. The right picture shows the completely resected mass, whose pathological findings were compatible with the diagnosis of teratoma.

Chest X‐ray demonstrated a space‐occupying mass in the right hemithorax, resulting in mediastinal shift to the left (Figure 2). CT imaging suggested teratoma, and the lesion was categorized as HITM based on our proposed classification (Figure 2). A sono‐guided core needle biopsy confirmed the diagnosis of teratoma.

### 3.2 Differential Diagnosis

Teratoma, Thymic tumor, Germ cell tumor, Bronchogenic cyst.

### 3.3 Outcome and Follow‐Up

The patient underwent a right posterolateral thoracotomy. As the inferior border of the mass was free, the fourth intercostal space was initially identified; however, entry into the thoracic cavity through this space was not possible. To obtain adequate access, the fourth rib was resected. Severe adhesions were observed between the mass and the hilar structures of the lung, including the pulmonary artery and bronchi. These adhesions were carefully released. Following complete release, the mass was removed. A chest tube was then inserted, and the thoracotomy was closed. The operative time was 4 h. No blood transfusion or postoperative complications were observed.

Postoperatively, the patient was monitored in the ICU for 24 h before being transferred to the ward. She was discharged in good condition 1 week after surgery.

## 4 Case 3

### 4.1 Case History

A 71‐year‐old man presented to our hospital with progressive shortness of breath and intermittent coughing. On admission, his vital signs were: pulse rate (PR) 92/min, blood pressure 140/85 mmHg, respiratory rate (RR) 28/min, and temperature 36.8°C. On physical examination, he appeared cachectic and exhibited rapid, shallow breathing. The left hemithorax showed no movement with respiration, and breath sounds were absent on that side. Laboratory testing revealed a white blood cell (WBC) count of 8000/μL and a hemoglobin level of 13.1 mg/dL.

**FIGURE 3:**
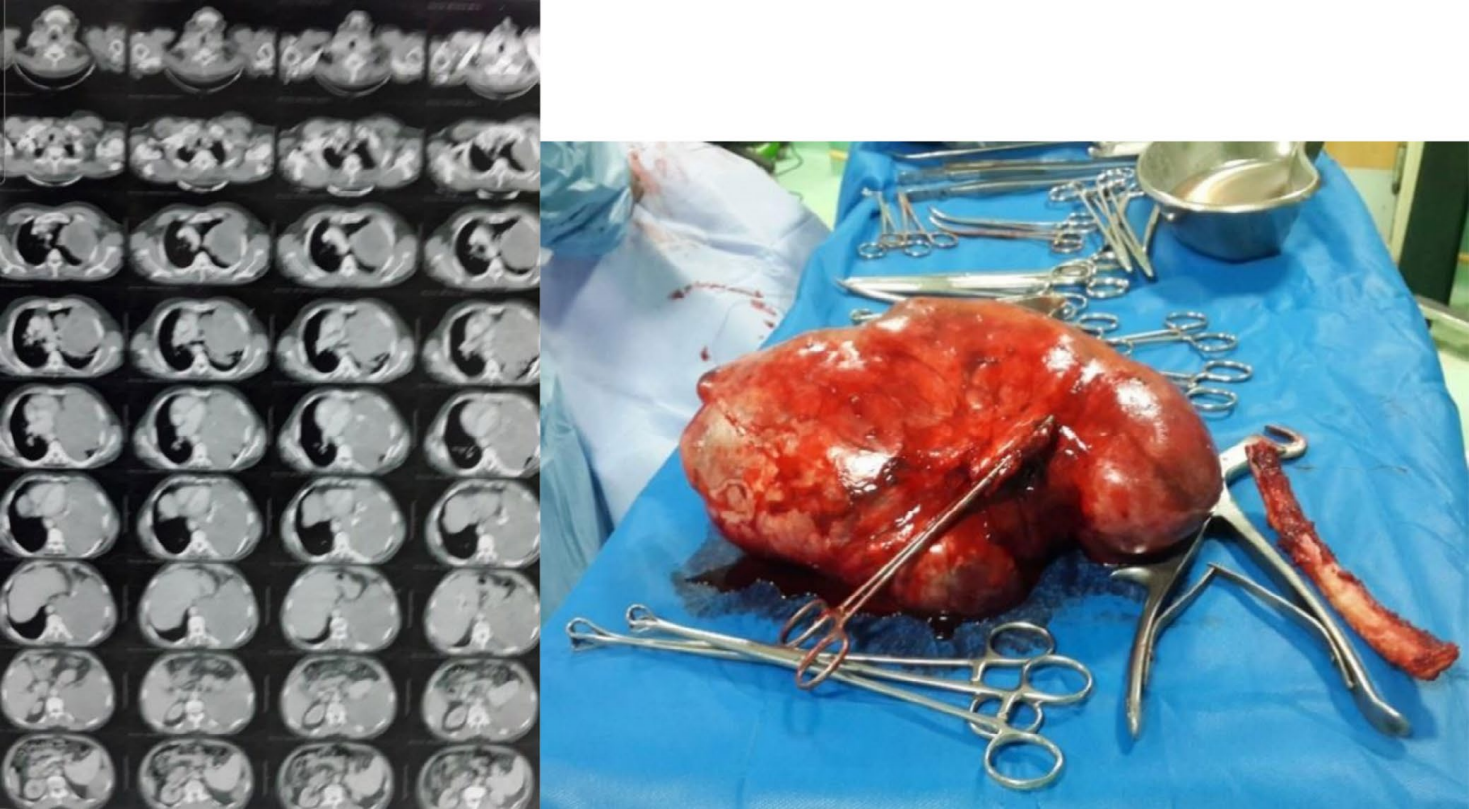
Left, lung CT scan shows a huge intrathoracic mass, which was extracted completely (right picture) by our method of surgery. The pathologic findings confirmed the diagnosis of a solitary fibrous tumor.

Chest X‐ray demonstrated a large space‐occupying mass in the left hemithorax, resulting in mediastinal shift to the right. A CT scan confirmed the presence of a huge intrathoracic mass categorized as HITM (Figure 3). Sono‐guided core needle biopsy was compatible with a solitary fibrous tumor.

### 4.2 Differential Diagnosis

Solitary fibrous tumor, Pleural mesothelioma, Fibrosarcoma, Sarcomatoid carcinoma.

### 4.3 Outcome and Follow‐Up

A left posterolateral thoracotomy through the fourth intercostal space was performed. However, entry into the thoracic cavity was not possible through this space. As the superior border of the mass was free, the fifth rib was resected to facilitate access. The attachments of the mass to surrounding tissues were released, with most adhesions located at the mediastinal pleura. These adhesions were ligated using silk sutures. After complete excision of the mass, a chest tube was inserted, and the thoracotomy was closed. The operative time was 3 h. No blood transfusion was required. The postoperative course was uneventful, except for a transient rise in serum creatinine, which resolved with conservative management.

Postoperatively, the patient was monitored in the ICU for 24 h and was subsequently transferred to the ward. He was discharged after 1 week in good general condition.

## 5 Discussion

Giant intrathoracic tumors present considerable diagnostic and surgical challenges due to their size, adhesions to surrounding structures, and impact on respiratory and mediastinal anatomy [7]. Reports in the literature describe a range of tumor types, such as teratomas, solitary fibrous tumors, lipomatous tumors, and primary chest wall sarcomas, that may occupy large portions of the hemithorax and cause displacement of intrathoracic organs [6, 8]. Across these studies, the most frequently cited intraoperative difficulties include limited access, dense adhesions, risk of bleeding, and difficulties in maintaining en‐bloc resection without fragmentation [9, 10].

Multiple surgical approaches have been documented for managing giant thoracic masses. Video‐assisted thoracic surgery (VATS) has been used in selected cases, particularly where tumors are well‐encapsulated and vascular control is feasible [11]; however, most published series emphasize that minimally invasive techniques are generally unsuitable for lesions with extensive pleural or mediastinal involvement, or when the tumor occupies the majority of the hemithorax. In such situations, open thoracotomy remains the preferred approach [6]. Variations such as extended posterolateral thoracotomy, double‐level thoracotomy, and clamshell incisions are reported to improve exposure and facilitate safe dissection, particularly when the mass adheres to the mediastinum, chest wall, or diaphragm [8, 12].

For tumors with chest wall involvement, including malignant lesions such as sarcomas, wide resection with rib removal and reconstruction may be required to ensure adequate margins and restore structural integrity [13]. Published cases highlight the use of meshes, titanium plates, or combined techniques to prevent chest wall instability and protect underlying organs. Achieving en‐bloc resection has been associated with better local control and reduced recurrence risk, although it often necessitates complex planning and interdisciplinary support [14, 15]. In our series, rib resection was performed subperiosteally in all cases. In selected cases with chest wall involvement, the primary rib along with adjacent ribs was resected to ensure adequate margins. Although extensive chest wall reconstruction was not required in our patients, multiple rib resection with immediate reconstruction is feasible, particularly in adults. Recent studies have demonstrated favorable outcomes using biocompatible implants, including titanium‐ and TiNi‐based systems, for chest wall reconstruction [16, 17].

Several studies also recommend adjunctive strategies for large or hyper‐vascular tumors, including preoperative embolization, neoadjuvant chemotherapy in malignant cases, staged resections, and perioperative intensive care planning. These measures aim to minimize intraoperative bleeding, facilitate dissection, and improve postoperative recovery [18, 19, 20].

Our series contributes to the growing body of literature in three key ways. First, it demonstrates that giant intrathoracic tumors of varying pathologies can be completely resected with favorable early outcomes when surgical access is carefully planned and fragmentation is avoided. Second, the intraoperative difficulties we encountered, such as the inability to enter through the standard intercostal space, extensive adhesions to mediastinal or hilar structures, and the need for rib resection, are consistent with previously reported challenges, underscoring the importance of adaptable strategies. Third, we applied our proposed classification, which categorizes tumors based on volumetric involvement of the hemithorax and the number of surfaces adhered to. This framework may help in preoperative planning by distinguishing tumors that can be approached through standard thoracotomy from those requiring modified access.

**FIGURE 4:**
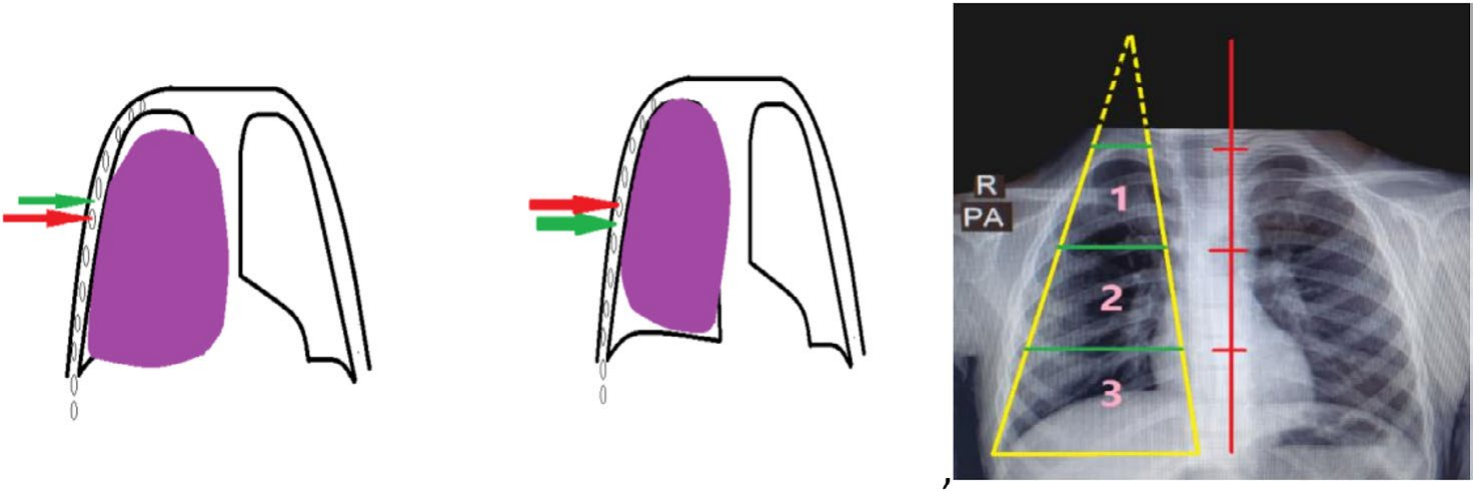
Truncated five‐sided pyramid model and target rib selection and access plan.

In this study, we conceptualize each hemithorax as a truncated five‐sided pyramid, with the diaphragm forming the base and the anterior, posterior, lateral, and medial surfaces representing the other sides. Based on volumetric assessment from chest CT, intrathoracic masses are categorized into five groups: small, medium, large, very large, and huge according to their proportion of hemithorax volume and surface involvement (Table 1). Small masses (≤ 10%) may be amenable to thoracoscopic resection, while medium to very large masses can be approached via conventional thoracotomy. Huge intrathoracic masses (HITM), defined as those occupying > 50% of the hemithorax and adhering to three or more surfaces, present significant challenges for safe exposure and en‐bloc resection. For these lesions, our surgical approach identifies the “target rib” adjacent to the free edge of the mass; resection of this rib enables entry into the thoracic cavity while preserving surrounding neurovascular structures. When no free edge is present, the resection is planned at the most voluminous portion of the mass, allowing complete removal without fragmentation (Figure 4). This classification and approach provide a systematic framework for preoperative planning and tailored surgical access for complex intrathoracic masses.

**TABLE 1:** The new classification according to the mass volume in relation to the hemithorax cavity and surface involvement.

Small masses	≤ 10% of the hemithorax volume
Medium masses	11%–30% of the hemithorax volume
Large masses	31%–50% of the hemithorax volume
Very large masses	51%–80% of the hemithorax volume
Huge masses (HITM)	(A) 51%–80% of the hemithorax volume and adhesion to three or more sides of the hemithorax pyramid
	(B) ≥ 80% of the hemithorax volume

In our case selection, CT imaging identified tumors with adhesion to adjacent structures but no evidence of invasion, allowing en‐bloc resection in all cases. Our classification is based on tumor volume and surface involvement and does not account for invasion. In malignant tumors with invasion, additional surgical strategies may be required and en‐bloc resection may not be feasible. However, in benign tumors such as teratomas, despite dense adhesions, our approach remained effective with meticulous surgical technique.

In cases where the tumor occupied most of the hemithorax or had no easily accessible free margin, our surgical method enabled safe entry by identifying ribs adjacent to the mass, resecting the rib that allowed optimal access, and then releasing the tumor from the free border. This facilitated en‐bloc removal and avoided fragmentation, which is desirable for reducing seeding risk and maintaining oncologic control. In the cases requiring rib resection and chest wall repair, reconstruction with synthetic mesh yielded satisfactory early outcomes.

In addition to achieving oncologically adequate margins, resection of adjacent ribs may improve surgical exposure and facilitate safer en‐bloc resection, particularly in large or complex lesions. This technical consideration is especially relevant when dealing with tumors extending toward mediastinal or intrathoracic structures [21].

In our approach, routine proximal vascular control was not performed. Instead, vascular structures were managed through meticulous dissection under direct vision following adequate exposure achieved by targeted rib resection. This allowed safe handling of mediastinal attachments without major vascular complications in our series. However, in cases with suspected vascular invasion or high bleeding risk, more extensive approaches such as median sternotomy or clamshell thoracotomy may be required.

We acknowledge limitations such as small sample size and limited follow‐up. Future research with larger cohorts and longer observation is needed to evaluate recurrence risk, functional outcomes, and the broader applicability of this approach. Comparative studies examining standard thoracotomy versus targeted rib resection in the setting of giant intrathoracic tumors may further clarify the advantages and limitations of this technique. Our series included only benign or low‐grade tumors; therefore, the applicability of this approach to aggressive malignant sarcomas remains uncertain.

In summary, complete resection remains the principal therapeutic strategy for large intrathoracic tumors, and existing literature supports open approaches when the mass occupies most of the hemithorax or adheres to multiple surfaces. Our proposed classification offers a framework for assessing tumor extent and surface involvement, while our surgical method provides a practical approach for achieving safe exposure and en‐bloc resection in cases where conventional thoracotomy is insufficient. These contributions may assist surgeons in operative planning and improve outcomes in patients with giant intrathoracic masses.

## 6 Conclusion

Huge intrathoracic masses require careful preoperative planning and modified surgical access. Our classification system and targeted rib resection technique enabled safe entry and en‐bloc tumor removal in all three cases. This approach may improve surgical outcomes when conventional thoracotomy is insufficient.

## Author Contributions

**Mohammad Reza Farahnak:** conceptualization, data curation, investigation, writing – original draft, writing – review and editing. **Muhammad Sina Salimikouchi:** conceptualization, methodology, validation, writing – review and editing. **Mohammad Javad Najafzadeh:** conceptualization, writing – original draft, writing – review and editing. **Amir Baniasad:** conceptualization, investigation, methodology, writing – original draft, writing – review and editing.

## Funding

The authors have nothing to report.

## Ethics Statement

This case series was conducted in accordance with institutional and national ethical standards. Ethical approval was granted by the Ethics Committee of Ahvaz Jundishapur University of Medical Sciences under the code IR.AJUMS.REC.1403.204.

## Consent

Written informed consent was obtained from all patients for publication of their clinical details and images.

## Conflicts of Interest

The authors declare no conflicts of interest.

## Data Availability

Data supporting the findings of this study are available from the corresponding author upon reasonable request.
